# Push and pull factors influencing the choice of a health resort by Polish treatment-seekers

**DOI:** 10.1186/s12889-023-17086-5

**Published:** 2023-11-08

**Authors:** Agnieszka Godlewska, Anna Mazurek-Kusiak, Andrzej Soroka

**Affiliations:** 1 Faculty of Medical and Health Sciences, University of Siedlce, B. Prusa 14 st., 08-110 Siedlce, Poland; 2 Department of Tourism and Recreation, University of Life Sciences in Lublin, Akademicka15, Lublin, 20- 950 Poland

**Keywords:** Motivation, Health tourism, Destination choice, Additional services, Tourism

## Abstract

**Background:**

The objective of the study was to indicate social and psychological (inner) motives associated with the desire and needs of travelling to health resorts (push) as well as external motives resulting from destination’s attributes, and explaining the choice of the health tourism establishment (pull) by Polish treatment-seekers divided into two social groups: persons in employment and retired persons. The research hypothesis assumed the two groups of people differ very much in terms of preferences motivating them to participate in health resort tourism, destination choices and services offered by health resort establishments.

**Methods:**

Of the 258 health resorts, 154 establishments were selected for research purposes taking into account a proportional distribution of health resorts throughout Poland. An authorial questionnaire was developed and it included three questions with multiple answers, each answer being assessed on a 5-point Likert scale. The research adds new insights by analysing motives associated with health resort tourism in terms of benefits sought by visitors. The main and strongest motive for choosing health resort tourism is concern for health. In addition to old age pensioners, it was legitimate to analyse the group of employed respondents as their stays at a health resort are undertaken to regain not only physical fitness but also work motivation.

**Results:**

The present study has demonstrated that motives and age groups have a significant effect on destination choice. Based on the results, it can be concluded that these groups often have diverse needs, expectations and, as a result, motives for choosing this form of tourism and a given health resort. What follows is a possibility of dividing the visitors to health resorts in terms of push and pull motives.

**Conclusions:**

The research hypothesis assuming the two groups varied considerably in terms of preferences motivating them to participate in health resort tourism, destination selection and choice of services offered by the health resort establishment has been confirmed. The ramifications of the study’s findings may also be relevant for health resort/spa tourism outside of Poland because healthy lifestyle promotion is a worldwide trend.

## Introduction

Increasing wealth and health awareness of society, changing lifestyles, and globalisation have contributed to tourism development worldwide [1]. Travel destination choices are very much affected by the form of tourism one prefers. Diseases of civilisation, a stressful way of life on the one hand, and healthy lifestyle on the other hand are factors affecting health tourism which has become one of the most rapidly growing segments of tourism industry [2–4]. It is a market with perspectives which will continue to develop in the years to come [5]. Such a tendency indicates there is demand for health improvement options [6], relaxation [7] and stress alleviation [8] by consuming health-related services.

Health tourism directly improves the tourist’s health condition and indirectly positively affects his or her general well-being in social, physical, mental and spiritual terms [9]. Health tourism includes health resort tourism which is offered at tourist health resort establishments. Its aim is to provide curative treatment services, including treatment of chronic diseases, rehabilitation, prophylaxis, education and health promotion [10].

Health resorts are dedicated establishments which provide curative treatment services using natural resources with medicinal properties [11]. Poland has 45 licenced health resorts which may receive tourists seeking treatment and biological renewal in addition to interesting tourist assets. Tourist attractiveness combined with modern infrastructure renders Polish health resorts one of the most attractive tourist destinations in Europe [12] which still offer traditional treatment facilities associated with balneotherapy or climate therapy [13].

Tourists seeking treatment and additional services endeavour to retain good health and prevent diseases by spending their time in a relaxing and natural environment thus benefiting from the very health resort facilities and its surroundings [14]. Health tourism is increasingly associated with other forms of tourism such as sports, recreation or culture [15, 16]. It is a combination of active relaxation, prophylaxis and treatment [17]. The development of health tourism is accompanied by changing needs and motives behind tourists’ choice of destination. Therefore, motives which prompt people to travel for health tourism-related purposes, including health resort tourism, vary and are affected by a variety of factors. Therefore, the authors of the present work attempted to conduct research which aimed at indicating motives associated with the desire and needs of travelling to health resorts (push) and explaining the choice of the health tourism establishment (pull) by Polish treatment-seekers divided into two social groups: persons in employment and retired persons. The research hypothesis assumed the two groups of people differ very much in terms of preferences motivating them to participate in health resort tourism, destination choices and services offered by health resort establishments.

The contribution of the present work to the theory and practice consists in adding to the literature on health tourism with insight into push and pull motives of a group of tourists. Moreover, analysis of motive segmentation based on Polish health resorts, to some extent will add information and make it possible to evaluate tourist demand for European health resorts. However, one should bear in mind there are some limitations resulting from e.g. differences in financial circumstances of older people from different countries and different subsidizing methods by the state.

## Literature review

Motivation is a psychological process which influences human behaviour and actions in order to achieve the assumed aim [18, 19]. It is driven by human needs (internal and external forces).

The effect of selected motives on destination choice by tourists and their decision to revisit the destination have been studied by Ćulić et al. [20] and Gajić et al. [21]. Tourist motivation is an integration of biological and cultural forces affecting behaviour and experience as well as destination choice [22] which is conditioned by e.g. high quality of services offered to tourists or stable economic and political relationships affecting safety [23].Every motive which leads to travel falls into two universally accepted dimensions of motivation which are highly correlated push and pull factors. First publications on motivation in tourism appeared many years ago and included works by Plog [24], Dann [25], Beard, Ragheb [26, 27] and Virdi and Traini [28].

One of the most well-known theories of tourist motivation is Crompton’s concept [29] called ‘push-pull’ [30]. It assumes that people travel because of socio-psychological (internal) motives which were drivers (pushed them) to travel (pull). Push motives include recovery after an illness or injury, removal of negative results of stress, addiction treatment, the decision to improve one’s health or health prophylaxis [31, 32]. External (pull) motives associated with destination’s attributes explain the choice of destination and they include: expectations, benefits, travel perception or attributes of the destination [33, 34]. The choice of destination is very much conditioned by other motives related to individual backgrounds, societies, occupational groups or age groups [16, 35] as well as stereotypes and a tourist’s prejudices against visiting a given destination [36]. The so-called push-pull theory was applied to motives for the first time by Dann [37] while examining motives associated with holiday tourism. Push factors precede pull factors but one has to bear in mind that they are closely interrelated [25, 33]. Many authors have applied the push-pull theory in their works on tourist motivation [31, 38–40]. Both pull and push factors may pertain to different tourism forms and become a motive for travelling to get to know and improve the knowledge of heritage and culture [41], relax and escape from everyday life [42, 43], spend time in nature [44, 45] educate [43] search for comfort [46]. The need to stay healthy and prevent disease as well as health promotion are one of the oldest tourism-related motives [47].

In health tourism, tourists are motivated by prophylaxis or treatment goals to improve their health and/or well-being [48]. Research is also conducted into motivation behind participation and choice of services offered by health tourism [49–55]. It suggests that an understanding of the motive a tourist is driven by is of importance for tourism development. Baliga [56] lists motives which imply choosing this form of health tourism. They include:

- Recovery after illness or injury,
- The need to remove negative effects of stress,
- Anti-aging and beautifying procedures (including plastic surgery),
- Addiction rehabilitation,
- The decision to improve one’s health by using specialist health care,
- Intervention or activity in a laid-back health resort atmosphere unlike a hospital setting,
- A way of an access to increasingly variable complementary therapies associated with prophylaxis of health protection means.

The literature on the subject of health tourism provides examples of different approaches to selecting or dividing factors which motivate visitors. Some authors examine motives in health tourism without referring to pull and push motives [49, 57–60]. Analysis of motives taken into consideration in health tourism reveals few works in which motives are divided into push and pull factors [53, 58]. A groups of pull motives behind health tourism was described by Chen et al. [61], Dryglas and Salamaga [62], and Mueller and Lanz Kaufmann [60]. The majority of researchers have considered only push factors in their works [13, 59, 63–67].

In their work, the authors refer to push motives is different ways, e.g.:

- Receiving medical treatment, visiting friends and relatives plus receiving medical treatment, exploring new places and cultures plus receiving medical treatment, fulfilling a dream plus, receiving medical treatment, reasons other than receiving medical treatment [68],
- Desires to participate in employment, physical activity, and daily life, determination to diminish bodily pain, desires to overcome perceived or experienced lack of access to care at home [67],
- Self-development, health and physical activity, relaxation and escape, isolation and nostalgia, nature, autonomy and stimulation, and social status [59].

Also pull motives are viewed differently by authors:

- Tangible resources and marketing image [53],
- Personnel services, health promotion treatments, environments, healthy diet, relaxation, social activities, experience of unique tourism resources, ental learning [61],
- Natural resources, cultural and natural environment, spa/wellness infrastructure and social and political environment [62].

All in all, in literature there is no universal and homogenous approach to push and pull motives of health resort visitors. Thus, it seems warranted to develop a pattern (method) of conducting this type of research.

## Methods

### Study population

The study involved a diagnostic survey method using a questionnaire. The method is frequently utilised in studies in the field of tourism and hotel industry. It was selected because it offers a possibility of examining a larger population and ensures the reliability of the method [69–71].

The research was conducted from April to October 2022, and it included 2,864 correctly completed questionnaires filled in by adult treatment-seekers and tourists. During the study period, the respondents stayed at health resorts for a period of at least 10 days, which guaranteed objectivity of questionnaire completion. The period of 10 days was assumed to be sufficient to fully get acquainted with the resort’s functioning, evaluate the level of treatments and personnel qualifications as well as asses factors which, according to respondents, should be changed. The validity of this assumption is supported by the very idea of treatment at Polish health resorts which offer stays from 2 to 3 weeks long, in some cases extending to as many as 26 days [72]. The analysis excluded 117 questionnaires due to incorrect completion.

Of 2,864 respondents, 120 (4.2%) were either pupils or university students, 991 (34.6%) were persons in employment, and 276 (9.6%) were either disability pension recipients or unemployed people. The most numerous group, 1,447 people (51.6%), consisted of retired persons. The research reported here included the two largest groups of respondents, that is persons in employment and retired persons. The main assumption of this research procedure was to distinguish people in employment, who need rest, regeneration and treatment before returning to the job, and a group of old age pensioners who already retired from their job.

### Research procedure

The procedure of study sample selection was based on the number of health resorts operating in Poland, located at 45 localities. As of December 2021, the number, which was used to calculate the sample size, equated to 258 [73]. Only fully residential health resorts were considered. The 95% confidence level was assumed while deciding on the study sample size, both fraction size and the maximum error being 0.5. A total of 154 establishments were agreed upon to be included the survey taking into consideration their proportional distribution all over the country.

To follow the established study protocol which required a 10-day stay, the target population was made up of only treatment-seekers and tourists choosing to stay at fully residential health resort establishments offering on-site accommodation. The number of people going to stay at such health resorts, as of December 2021, amounted to 529.9 thousand visitors of which 61.5% were females and 38.5% males [73]. Taking the above into consideration, as well as the number of working persons and pensioners, the overall number of questionnaires to be completed at each establishment was 16, with 10 taken by female respondents and 6 by male respondents, also in this group, 10 completed by pensioners and 6 by working persons, to account for the proportion between both sexes and social status. These two assumptions were the basis of selection of participants.

The study conforms to the code of ethics of the World Medical Association and the standards for research involving human subjects set out in the Declaration of Helsinki. The protocol was approved by the Ethics Committee Siedlce University of Natural Sciences and Humanities No. 2/2020. This research was performed following relevant guidelines and regulations. After an explanation of the aims and protocol of this study to each participant, written informed consent was obtained. The survey was conducted in a direct manner and was preceded by assuring each respondent about maintaining confidentiality.

### Data collection

As there is no homogenous division of pull and push motives, based on literature on the subject [13, 53, 58, 61, 62, 74, 75], an authorial questionnaire was developed. Motivation elements were divided into push (social and psychological motives) and pull (resulting from destination’s attributes, and explaining the choice of the health tourism establishment) factors. An questionnaire was developed with three questions (one pertaining to push motives and two associated with pull factors) to which multiple possibilities of response were offered. Each answer was assessed on a 5-point Likert scale ranging from 1 (strongly disagree) to 5 (strongly agree). The survey was the main and only source of data obtained from tourists staying at health resorts.

The research objectives were achieved by analysing responses provided by participants of which:

- 20 answers pertained to socio-psychological motives (push),
- 15 answers (pull) explained choice of health resort establishment,
- 10 answers concerned additional services offered by the establishment and taken into consideration by tourists/treatment-seekers.

If an answer pertaining to the following push motives:

- The need to improve body capacity and fitness,
- The need to undergo procedures unavailable where one lives,
- The need to recover from an addiction developed as a form of relaxation,
- The need to improve one’s build to be more attractive;

or the following pull motives:

- The need to feel one is a fuller participant of the society,
- Reasonable prices at a given establishment,
- Location of the establishment within an urban agglomeration,
- Organisation of free time, diagnostic tests conducted both at the beginning and during the stay.

did not find favour with respondents or was viewed as unimportant, it was excluded from the created discrimination function models.

A preliminary survey, including 124 treatment-seekers and tourists, was conducted to validate the questionnaire and ensure the respondents found it clear and understandable. In this survey, internal validation of the instrument was performed, and the design was adjusted accordingly in order to obtain the best results. In the internal validation of the questionnaire, a Cronbach’s Alpha value of 0.876 was obtained, so demonstrating the reliability of the instrument, which scored well over the recommended ideal value of 0.7.

### Statistical analysis

STATISTICA 13 PL (TIBCO Software, PaloAlto, CA, USA) software and discriminant function were used to conduct statistical analysis. Discriminant analysis is a statistical method used to examine differences between three groups (such as in the study reported here) by a simultaneous analysis of several variables to find out which variables are the greatest contributors to group discrimination. Classification of cases is one of the main goals behind an application of discriminant analysis. The applied classification function is based on a linear combination of discriminant variables. There were two functions, the same number as the number of groups. They are used to determine to which group a given case belongs. Multidimensional normality was examined before testing, checking each variable for normality of distribution. We used the Kołmogorow-Smirnow test which verified the hypothesis that two samples were taken from different populations. Due to a large number of individuals in each group, slight deviations were of little importance [76]. It was assumed that the variances of the variables were homogeneous in particular groups. The variance matrix was not taken into account due to the large numbers of respondents in individual groups. Statistical significance was defined as those differences for which the probability of randomness was less than *p* ≤ 0.05.

## Results

The created discrimination function model included 14 out 20 selected motives pertaining to the desire and need (push) of using health resort establishments expressed by the respondents (Table 1). Four factors with the highest values of classification function represented health-related issues. Health concern was by far the strongest motive for both the groups. The motive was significantly, at *p* ≤ 0.001, more often pointed to by old age pensioners than the group of treatment-seekers/tourists in employment. Doctor’s recommendations expressing the advisability of staying at a health resort establishment were the next most important motive which obtained high values of classification function. In this case, slightly higher values of the classification function were found for the employed vs. retired group. The opportunity to lose weight and receive proper rehabilitation after an illness were another important motives of staying at a health resort, them being more significant, at *p* ≤ 0.001, for the persons in employment.

**Table 1:** Socio-psychological motives (push) behind treatment-seekers/tourists choosing to stay at health resort establishments

Type of motive	Wilks’ lambda: 0.525 F = 10.932 *p* < 0.001*			Classification function	
	Wilks’ lambda	F value	*P* level	Persons in employment	Retired persons
Health concern	0.523	14.91	0.001*	3.773	4.272
Doctor’s recommendations	0.539	0.860	0.327	2.146	2.054
Desire to lose weight	0.567	3.328	0.068	2.083	1.875
Desire to receive proper rehabilitation after an illness	0.512	19.444	0.001*	2.025	1.611
Desire to stay in the open air	0.496	33.034	0.001*	2.034	1.513
Desire to alleviate the negative effects of stress	0.539	76.178	0.001*	1.974	1.077
Desire to improve self-esteem	0.528	27.457	0.001*	1.525	1.045
Desire of mood improvement	0.519	12.871	0.001*	0.706	0.367
Desire to improve the quality of life	o.528	8.276	0.001*	0.689	0.439
Desire to extend the lifespan	0.487	19.147	0.001*	0.644	1.193
Escape from everyday problems	0.512	65.373	0.001*	0.324	0.594
Desire to undergo treatment under good conditions	0.530	6.176	0.013*	0.364	0.195
Trend to stay at a holiday resort	0.516	28.154	0.001*	0.072	0.298
Desire to be in good company	0.563	8.823	0.003*	0.082	0.418
Constant				19.345	21.043

*- level of significant differences at *p* ≤ 0.05

The desire to spend time in the open air, manage stress, and improve self-esteem, mood and quality of life constitute the motives which were significantly more important, at *p* ≤ 0.001, for employed respondents. Lifespan increase and escape from everyday problems was significantly more important for retired visitors, at *p* ≤ 0.001. Employed treatment-seekers/tourists significantly more often, at *p* = 0.013, perceived stays at a health resort as an opportunity to undergo healing treatments and rehabilitation under favourable conditions. The final two factors which entered the created model were social factors, that is prevailing trend to enjoy a stay at a health resort and the desire to socialise with interesting people. Significantly higher values of classification function were obtained for retired respondents at *p* ≤ 0.001 for trends of staying at a health resort, and *p* = 0.003 for the desire to be in good company. The created discrimination function model did not include the desire to: improve the capacity and physical fitness of the body, undergo treatments unavailable at one’s place of residence, quit addictions, treat the stay as a form of relaxation, improve one’s figure and strengthen one’s conviction of fuller functioning in the society. The model of discrimination function created in this way demonstrated that not all the classified push motives were of importance to customers.

The created model took into account 12 out of 15 motives explaining destination choice (pull) (Table 2). By far, the most important motive was cleanliness and safety during the stay. Retired respondents paid significantly more attention, at *p* = 0.021, to this aspect of the stay. At *p* ≤ 0.001 and with higher values of classification function for old age pensioners, there was much variation associated with factors reflecting a high level of medical, treatment and diagnostic infrastructure, a wide range of rehabilitation, relaxation and treatment services on offer, and good gastronomic facilities. Also retired respondents, at *p* = 0.027, more often declared an importance of offering treatments which are not likely to produce any complications. The feeling of safety was to a great extent associated with the factor of quietness the visitors expected to enjoy while staying at a health resort, and an availability of sports equipment. In both the cases there were only slight differences between values of classification function created for the analysed groups of respondents. More often than retired visitors, employed participants, at *p* ≤ 0.001, wanted an attractive location of the health resort, and a high standard of gastronomic services and accommodation. Old age pensioners significantly more frequently, at *p* ≤ 0.001, mentioned the desire to stay within a forest-parkland complex and, at *p* = 0.008, the interest in an availability of cinema, theatre of shopping centre facilities in the surrounding area.

**Table 2:** Pull motives explaining health tourism destination choice

Type of motive	Wilks’ lambda: 0.525 F = 10.932 *p* < 0.001*			Classification function	
	Wilks’ lambda	F value	*P* level	Persons in employment	Retired persons
Cleanliness and safety at the establishment	0.398	5.301	0.021*	9.113	9.478
High level of medical, treatment and diagnostic infrastructure	0.389	13.046	0.001*	6.61	7.02
A wide array of rehabilitation, relaxation and treatment options	0.417	17.458	0.001*	5.444	4.934
Offering treatments which are not likely to produce complications	0.412	4.877	0.027*	4.367	4.628
Good gastronomic facilities	0.367	22.459	0.001*	2.705	3.304
Quietness at the establishment	0.388	2.426	0.119	2.887	3.067
Availability of sports infrastructure	0.392	2.940	0.086	2.198	2.307
Tourist attractiveness of the locality	0.412	30.657	0.001*	2.883	2.127
Availability of very good specialists	0.421	10.651	0.001*	1.765	2.187
Location within a forest-parkland complex	0.408	19.678	0.001*	0.867	1.329
Presence of infrastructure (cinema, theatre, shopping centre) in the surrounding area	0.431	6.987	0.008*	1.067	1.223
High level of accommodation and gastronomic services	0.396	24.408	0.001*	0.818	0.432
Constant				40.056	40.957

*- level of significant differences at *p* ≤ 0.05

Eight out of ten pull motives contained in the questionnaire entered the model of discrimination function, them reflecting additional services offered to visitors by health resort establishments (Table 3). The most important pull motive was adjustment of the offering to the relaxation, rehabilitation and curative needs of the customer. A significantly higher demand, at *p* ≤ 0.001, for this type of services was expressed by retired respondents who were also significantly more likely, at respectively *p* = 0.021 and 0.002, to require more free-of-charge initial and further diagnoses as well as an opportunity to obtain information leaflets/brochures about the establishment. Both the surveyed groups were similarly likely to decare the need for the health resorts to offer additional packages for accompanying persons and an opportunity to make use of promotional packages. Persons in employment significantly more often pointed to the need to use additional treatments (at *p* = 0.011), an opportunity do change treatments during the stay (at *p* = 0.037) and an unlimited availability of a doctor or physiotherapist to consult should a need arise (at *p* = 0.049).

**Table 3:** Pull motives associated with additional services at a health resort establishment

Type of motive	Wilks’ lambda: 0.525 F = 10.932 *p* < 0.001*			Classification function	
	Wilks’ lambda	F value	P level	Persons in employment	Retired persons
Adjustment of package to relaxation, rehabilitation and medical needs of the customer	0.453	16.687	0.001*	4.363	4.758
Free-of-charge initial and further diagnoses	0.459	5.302	0.021*	3.245	3.445
An opportunity to obtain information leaflets/brochures about the establishment	0.421	63.208	0.002*	2.665	3.136
Additional packages for accompanying persons on offer	0.408	2.130	0.144	2.434	2.325
An opportunity to make use of promotional packages	0.432	0.991	0.331	2.132	2.026
An opportunity to use additional treatments	0.454	6.373	0.011*	2.229	1.913
An opportunity do change treatments during the stay	0.473	4.043	0.037*	1.278	0.971
Unlimited availability of a doctor or physiotherapist to consult on request	0.452	3.793	0.049*	0.991	0.819
Constant				13.375	12.438

*- level of significant difference at *p* ≤ 0.05

## Discussion

Results of the survey made it possible to achieve the assumed objective which was to indicate motives both employed and retired persons have for staying at holiday resorts. Also hypothetical assumptions of the study revealed differences and changing preferences of motives between the major customers of health tourism.

The main motive which was also the most strongly accentuated by both the groups of respondents was health and health concern, which agrees with earlier reports by Araújo Vila et al. [77], Rodrigues, Brochado, Troilo [5] and Tuominen et al. [48]. The motive was significantly more appreciated by retired visitors who were older than respondents in employment, which seems understandable as age is a factor which generates more concern and care for one’s health [78].

It was demonstrated in the study that doctor’s recommendations were a powerful motive which patients followed, in particular whose who were in employment, which concurs with findings reported by Kucukusta, Guillet [79].

People in employment were much more significantly interested in body weight loss, which appeared to be one of more important motives pointed to by respondents. In recent years, body care services, including slimming, skin care and nicotine addiction treatment, have become a trendy direction of healthy lifestyle care [80].

Rehabilitation needs after an illness turned out to be an important motive, in particular for working persons, which seems understandable in view of their need for convalescence and rest. In the Polish healthcare system, employed people requiring rehabilitation in order to return to their usual work-related routine or not to reduce their working activity due to health-related reasons, may bid for a stay at a health resort which will be entirely paid for by the Social Insurance Institution (ZUS) within the ZUS disability prevention option. In order to be considered, the patient is required to supply a medical certificate stating there exists a need for rehabilitation [81].

Striving for well-being and having a pleasurable stay at a holiday resort, both being hedonistic factors [77], were strongly emphasised in the study, predominantly by employed participants. People travel to health resorts for health-related purposes [82, 83] although they are also in search of relaxation, happiness, rejuvenation and concern for their well-being [84, 85]. Alleviation of negative influences of stress, an improvement of mood and quality of life are also important motives behind people’s decision to participate in health tourism [86, 87]. Well-being, as demonstrated in the present work, is one of four main motives of health resort tourism [64, 88]. It is believed that the variables reflecting well-being and the feeling of pleasure are more important for younger visitors –working people in the case of the research reported here [89–91]. Also this group of treatment-seekers, as important motives, pointed to improvement of the quality of life, ease of mind and the feeling of relief, all of them associated with distancing from everyday work-related tasks [92] or everyday routine [53, 93].

Natural factors contributing to communion with nature, such as spending time in the open air surrounded by a beautiful landscape and greenery are important motives of participating in health resort tourism [16, 87]. The desire to prolong one’s life and escape from everyday problems was significantly more important for old age pensioners. Employed visitors expressed the desire to undergo treatments, both curative and rehabilitation, under good conditions, which was a declaration of a conscious need to take care of one’s health through a variety of treatments and, as demonstrated in the present study, using a wide range of such services on offer [92, 94].

The desire to be in good company reflects the need to renew the physical and emotional balance as well as update socialising, the lifestyle and social identity, all of which being expressed more often by retired visitors [95].

Of the motives explaining choice of a given health resort establishment declared by both groups of visitors, the most important ones included cleanliness and safety during the stay. Safety at the establishment was the element which contributed to the good mood of treatment-seekers [96] and satisfaction from the stay, which positively affected their well-being [97]. People attach increasingly more importance to safety at the establishment they are visiting but also during the journey to and from it [98, 99]. In turn, cleanliness is closely associated with expectations of visitors as to the services on offer such as a high level of medical, treatment and diagnostic infrastructure at the health resort in addition to a qualified personnel who follow the hygiene procedures, and a high quality of offered services [100].

The motive of the health resort establishment’s location within a forest-parkland complex or in a natural environment was significantly more appreciated by retired respondents. Such preferences had been previously reported in studies by Antunes and Cunha [80, 101], both pointing to the desire of treatment-seekers to relax in contact with nature [70, 79]. Hence, not only the very destination but also its surroundings seem to have become an important aspect [102].

In order to achieve the balance between body and mind, individuals satisfy their psychological needs by sports and relaxation activities [103], these aspects being mentioned by both groups participating in the survey reported here.

As of now, operation of health resort establishments is focused on offering both health- and leisure-oriented services. Due to this, it seems necessary to develop new products and services, which will be increasingly exclusive, creative and unique to draw visitors and make the health resort a one-of-a-kind establishment [104]. Striving for good health, well-being and healthy lifestyle will be increasingly more important for the Polish society, so the health tourism sector, including health resort tourism, will be constantly developing [105, 106].

As people, especially those who work, are constantly in a rash, they search for means of stress alleviation and taking care of their mental and physical health [107]. The peaceful and quiet atmosphere at the establishment contributes to stress alleviation, which leads to a healthy balance of body, mind and spirit, and results in a good mood of visitors [108]. This agrees with results of studies which point to a favourable perception of the surroundings. Such attitude creates a feeling of pleasure and it positively affects the subjective memories [109, 110]. Growing interest in a healthy lifestyle combined with physical and intellectual activity directly contributes to altered patterns of spending free time, including stays at holiday resorts [111]. For such change to take place, the whole process is necessary of informing patients about services available during the stay [112], which was indicated by respondents in the study reported here.

The society is displaying an increasing health awareness [113, 114]. What is important to treatment-seekers is not only the very stay at a health resort but also adjustment of the offering to the relaxation, rehabilitation and medical needs of the guests, with offerings including a free-of-charge access to initial and further diagnoses by doctors. The awareness that good health is the most important and valuable asset in life [115] results in treatment-seekers travelling to health resorts to enjoy their benefits [116, 117]. During the stay at a health resort, visitors want to make the best use of its offerings. This translates into the desire to enjoy additional services or to change them during the stay, as indicated by both the groups of respondents. Such arrangements lie with establishments. As a result, offering additional services, often free of charge, seems to have become a responsibility of health resort establishments [118–120].

Health resorts are recommended to consider an extensive improvement of aesthetic components of their facilities [121], and conditions affecting mental and physical relaxation, and an introduction of relaxation workshops [79]. Laureiro [122] claims the need exists to take care of entertainment such as cultural events and sightseeing in the surrounding area which, as confirmed in the present study, are taken into consideration while choosing a health resort, by retired persons in particular. However, Damijanić [123] has stated that cultural activity, parties and shopping are unimportant tourist motivations.

## Conclusions

Promoted worldwide healthy lifestyle underlies present-day social transformations in Poland. It results in a growing interest in and meaning of health tourism, including health resort tourism. There were indicated push and pull motives of tourists choosing health resort tourism and destination. An understanding of the motives behind people travelling to health resort establishments is of utmost importance for the development of this sector of tourism. The research complements and fills in gaps in research examining the strength of push and pull motives of people participating in health resort tourism, which contributes to better management of health resorts and creation of conditions meeting expectations of potential tourists. The research adds new insights by analysing motives associated with health resort tourism in terms of benefits sought by visitors. The main and strongest motive for choosing health resort tourism is concern for health. In addition to old age pensioners, it was legitimate to analyse the group of employed respondents as their stays at a health resort are undertaken to regain not only physical fitness but also work motivation. The study has demonstrated that motives and age groups have a significant effect on destination choice. It can be concluded that these groups often have diverse needs, expectations and, as a result, motives for choosing this form of tourism and a given health resort. What follows is a possibility of dividing the visitors to health resorts in terms of push and pull motives. The research hypothesis assuming the two groups of treatment-seekers varied considerably in terms of preferences motivating them to participate in health resort tourism, destination selection and choice of services offered by the health resort establishment has been confirmed. The ramifications of the study’s findings may also be relevant for health resort/spa tourism outside of Poland because healthy lifestyle promotion is a worldwide trend.

### Limitations and future research

It should be pointed out that the limitation of the study reported here as impossibility of extending the findings to other countries which is due to different financial circumstances of older people in, for example, Germany and Poland, or the methods of subsidizing the stays in question in various countries. Only pensioners and working people were included in the research reported here. It would be informative to extend future research to include other social groups. The limiting circumstance of this study was interaction with persons selected as respondents who, with no good cause, avoided participation. The large number of resorts to be included in the study (154) was also potentially a limiting factor as it required a substantial number of people to be involved as well as prior logistical examination. Another study will be undertaken with wider participation of other social groups and more issues associated with health resort tourism.

## Data Availability

The data supporting our findings are found at, kept in confidentiality and stored at the corresponding author both in hard and soft copies. If someone wants our data, we are voluntary to share it and the corresponding author should be contacted through the email address on the cover page.

## Declarations

research aim and methods before signing the informed consent form. The investigation conforms to the principles outlined in the Declaration of Helsinki.
